# Process design of microdomains with quantum mechanics for giant pulse lasers

**DOI:** 10.1038/s41598-017-10884-z

**Published:** 2017-09-06

**Authors:** Yoichi Sato, Jun Akiyama, Takunori Taira

**Affiliations:** Institute for Molecular Science, National Institutes of Natural Sciences, Okazaki, 444-8585 Japan

## Abstract

The power scaling of laser devices can contribute to the future of humanity. Giant microphotonics have been advocated as a solution to this issue. Among various technologies in giant microphotonics, process control of microdomains with quantum mechanical calculations is expected to increase the optical power extracted per unit volume in gain media. Design of extensive variables influencing the Gibbs energy of controlled microdomains in materials can realize desired properties. Here we estimate the angular momentum quantum number of rare-earth ions in microdomains. Using this process control, we generate kilowatt-level laser output from orientation-controlled microdomains in a laser gain medium. We also consider the limitations of current samples, and discuss the prospects of power scaling and applications of our technology. This work overturns at least three common viewpoints in current advanced technologies, including material processing based on magnetohydrodynamics, grain-size control of transparent polycrystals in fine ceramics, and the crystallographic symmetry of laser ceramics in photonics.

## Introduction

Recently, we proposed the concept of giant microphotonics (G-MiP)^1^. G-MiP generates very bright pulsed optical fields (giant pulses) from the microdomains in laser gain media using the extremely high stored energy realized by microchip laser (MCL) technology and the technology of controlled microdomain with quantum mechanical calculations (QC-MD). MCL is the generic term for laser devices that possess thin gain media (≪1 cm), and this technology enables the effective pumping and cooling of laser gain media because of their high aspect ratio. In other words, MCL technology can extend to the upper limit of the pulse repetition rate^2^. Meanwhile, QC-MD technology is the process design of the controlled microdomains. Materials composed of QC-MDs may display novel optical functions based on their artificial microdomain structures, which should enhance the extraction of optical energy per unit volume. While the repetition rate means the speed at which a laser works, the extraction energy density can show the upper limit of the inversion density in laser gain media, and describe the value of the work done by optical devices.

In reality, highly bright laser devices can achieve not only advances in pure physics (*e.g*., nonlinear quantum electrodynamics^3^), but also contribute to numerous human activities including experimental astronomy^4^, particle acceleration^5^, nuclear fusion^6^, heat engines^7^, diagnostics and environmental assessments^8^ and advanced machining^9^. The quality of laser devices should be improved so they can better contribute to the future of humanity. As an effective expedient, this work aims to realize QC-MD technology and confirm its advantages.

In this work, we synthesize orientation-controlled Yb^3+^-doped fluoroapatite Ca_5_(PO_4_)_3_F (Yb:FAP) with QC-MDs under a magnetic field ***B*** of 1.4 T. This synthesis represents the design of the Gibbs energy using QC-MD technology, and it generates kilowatt-level laser output.

The advantage of Yb^3+^ as a dopant is that its ionic radius is the shortest among RE^3+^, which allows Yb^3+^ to be substituted with ions in host materials more easily than other RE^3+^. Moreover, Yb^3+^ laser transitions display fewer quantum defects than other solid-state lasers^10^. Thus, doped Yb^3+^ can both enhance crystal magnetic anisotropy and activate lasing. We chose FAP as a host material in this work because of its potential as a high-energy laser gain medium with biocompatibility. Because of their large stimulated emission cross section *σ*_e_ and high thermal conductivity, apatite crystals including F^−^ in their crystal structure were originally proposed as laser host materials for the laser driver in the “Mercury Project”^11^, which aimed to increase the repetition rate of current laser drivers for inertial confinement fusion by 10^5^ times^12^. In addition, apatite crystals including Ca^2+^ in their crystal structure are known as raw materials with high biological affinity: they are components of bones and teeth^13^. If we establish Yb:FAP with QC-MD as a model case, a progressive *in vivo* laser device with highly bright output may be realized. Therefore, this work is also the starting point for the development of radical brain–machine interfaces for biomonitoring and assistance with neurological functions^14^.

This work overturns at least three common viewpoints in current advanced technologies. First, that extremely high magnetic fields of over 10 T generated by superconducting magnets are needed to align non-magnetic crystals^15^. Second, that grains smaller than the light wavelength are needed to suppress optical scattering in anisotropic materials^16^. Third, that only crystals with cubic crystalline symmetry are allowed to serve as raw materials for laser ceramics^17^. This work is not only a conceptual update of laser ceramics but also the first demonstration of optical materials with QC-MD technology.

## Results

### Process control of microdomains

Process control implies the elaboration of the magnetic anisotropy in a microdomain and of the orientation distribution of microdomains. The macroscopic status of a microdomain is described by its Gibbs free energy *G*. *G* is given by^18^

1 $$dG=-SdT+{\mu }dN+\frac{{\gamma }}{2}dA-V\sum _{ij}{{\varepsilon }}_{ij}d{{\sigma }}_{ij}-{\boldsymbol{P}}\cdot d{\boldsymbol{E}}-{\boldsymbol{M}}\cdot d{\boldsymbol{B}}{,}$$

where *T*, *γ*, *σ*_*ij*_, *ε*_*ij*_, ***E***, ***B***, *V*, *S*, *μ*, *N*, *A*, ***P*** and ***M*** are the temperature, boundary energy of domain surfaces, stress tensor, strain tensor, external electric field, applied magnetic-flux density, volume, entropy, chemical potential, number of molecules, surface area of the domain, dielectric polarization and magnetization, respectively. From the viewpoint of Equation (1), QC-MD is realized by the estimation of extensive factors like *ε*_*ij*_, ***P***, and ***M*** of microdomains with quantum mechanical calculations.

Even though current laser ceramic technologies modulate the first three terms on the right side of Equation (1), we directly controlled ***M***. In general, ionic crystals without open-shell electrons are diamagnetic when there are no electrons with non-zero angular momentum in their ground states. When trivalent rare-earth ions (RE^3+^) are doped into these materials, their paramagnetic characteristics are determined only by the doped RE^3+^ that possess unquenched azimuthal quantum number *L*. This means that we can design the crystalline magnetic anisotropy *Δχ* of the host crystals by doping with RE^3+^, where *Δχ* is the difference of magnetic susceptibility *χ* = *μ*_0_ ∂***M***/∂***B*** between ***B*** parallel to the *c*-axis and ***B*** parallel to the *a*-axis of the host and *μ*_0_ is the magnetic permeability in vacuum. Because of their large spin–orbit interaction, the state of RE^3+^ can be expressed by |*J*, *J*_z_ > where *J* and *J*_z_ are the total angular momentum of 4 *f* electrons in RE^3+^ and the component of *J* along the applied magnetic field, respectively. Using the set of |*J*, *J*_*z*_> , the energy levels of RE^3+^ are defined as eigenstates of the crystal field potential *V*_cryst_, which is given by^19^

2 $${V}_{{\rm{cryst}}}=-\frac{{e}^{2}}{4\pi {\varepsilon }_{0}}\sum _{l=2,4,6}\sum _{m=0}^{l}{(-1)}^{m}\sqrt{\frac{(l-m)!}{(l+m)!}}\frac{{\theta }_{J}}{{\lambda }_{l}^{m}}{f}_{l}^{m}\langle {r}^{l}\rangle {\hat{O}}_{l}^{m},$$

where *e*, *ε*_0_, *θ*_*J*_, *λ*_*l*_^*m*^, *f*_*l*_^*m*^, <*r* ^*l*^> and *Ô*_*l*_^*m*^ are the elementary charge, vacuum permittivity, Stevens factor of RE^3+^, normalization factor, lattice sum, expectation of the *l* ^th^ power of ionic radii and Stevens equivalent operator, respectively. We can estimate the anisotropy of 4 *f* electrons in RE^3+^ from *f*_*l*_^*m*^ calculated under the spatial coordination assigned to appropriate crystal axes.

**Figure 1:**
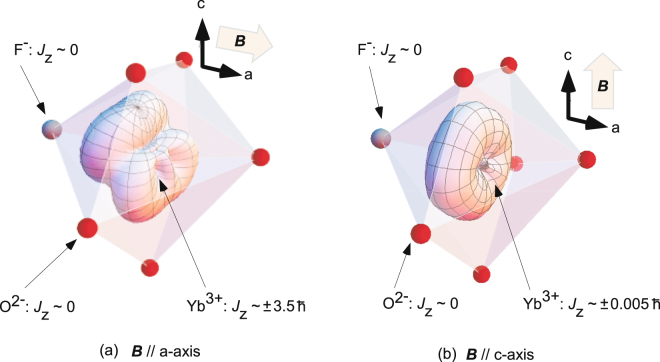
Dependence of the shape of Yb^3+^ in the crystal field on the direction of an applied magnetic field. Yb^3+^ is surrounded by one F^−^ (gray spheres) and six O^2−^ ions (red spheres), which is the same as the 6 *h* site in FAP (bold arrows indicate the crystal axes of FAP). The dependence of the shape of Yb^3+^ in the crystal field on the direction of the applied magnetic field causes the anisotropy of the total angular momentum of 4 *f* electrons in RE^3+^ along the applied magnetic field *J*_z_.

For example, we consider the case of the ground state of Yb^3+^ (Russell–Saunders term of^2^ *F*_7/2_) in a crystal field composed of seven ligands (six oxygen ions and a fluorine ion) like the 6 *h* site in a FAP crystal. As shown in Fig. 1, the shape of the electron cloud in this crystal field under ***B*** depends on the direction of ***B***. If ***B*** is parallel to the *c*-axis of FAP, the expected value of *J*_z_ of Yb^3+^ is *ca*. ± 0.005 *ħ*, where *ħ* is Planck’s constant. Similarly, *J*_z_ of Yb^3+^ is *ca*. ± 3.5 *ħ* when ***B*** is parallel to the *a*-axes of FAP. Other ions with only closed electronic shells (Russell–Saunders term of^1^ *S*) cannot provide paramagnetic *Δχ* because of their zero angular momentum.

Considering Kramers’ degeneracy, the second-order perturbation of the Zeeman effect gives ***M*** along the direction of ***B*** in one Yb^3+^ by^20^

3 $${\boldsymbol{M}}=\frac{{({g}_{J}{m}_{B})}^{2}}{kT}{\boldsymbol{B}}\sum _{i\in {}^{2}F_{7/2}}\langle i|{{J}_{Z}}^{2}|i\rangle {f}_{i}+2{({g}_{J}{m}_{B})}^{2}{\boldsymbol{B}}\sum _{i,j\in {}^{2}F_{7/2}}\frac{{|\langle j|{J}_{Z}|i\rangle |}^{2}}{{E}_{j}-{E}_{i}}{f}_{i},$$

where *g*_*J*_, *m*_*B*_, *k*, *f*_*i*_ and <*j*|*A*|*i*> are the Lande g-factor, Bohr magneton, Boltzmann constant, Boltzmann factor for level *i* and matrix element of an operator *A* between *i* and *j* states, respectively. Without any crystal field, Equation (3) simplifies to the Curie law, which indicates that doping with Yb^3+^ forms *Δχ*. As shown in Equation (3), Δχ depends not on *B* but on *V*_cryst_. In the case of the 6 *h* site in FAP, *a*- and *c*-axes are assigned as the easy- and hard-magnetization axes, respectively, because *Δχ* becomes negative.

### Orientation control with designed *Δχ*

**Figure 2:**
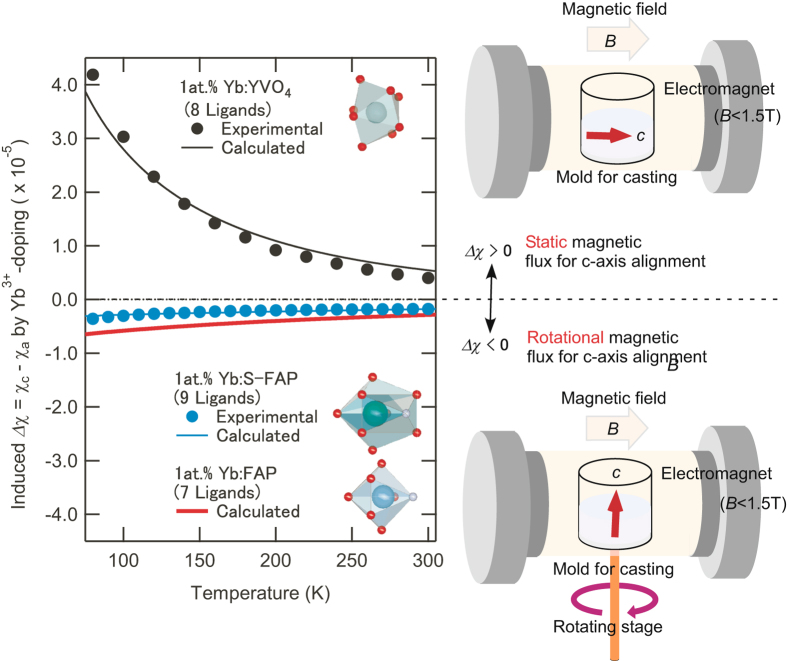
Calculated and experimental crystalline magnetic anisotropy *Δχ* induced by 1 at. % Yb^3+^-doping. If *Δχ* > 0, the orientation control of the *c*-axis in crystalline microparticles can be processed using a static magnetic field. Conversely, if *Δχ* < 0, orientation control requires a rotating magnetic field. A relative rotating magnetic field can be formed by rotating a slip-casting mold under a static magnetic field.

To limit the intensity of the applied magnetic field to the maximum that traditional electromagnets can generate (1.4 T), we enhanced *Δχ* by doping with RE^3+^, because *Δχ* of *N* times enables reduction of ***B*** by 1/*N* ^0.5^ times^20^. Figure 2 shows the calculated and experimental values for *Δχ* induced by Yb^3+^ doping for several anisotropic laser host crystals. We estimated that the induced *Δχ* for 1.6 at.% Yb:FAP was −4.6 × 10^−6^, and its absolute value was larger than that of Yb^3+^-doped strontium fluoroapatite Sr_5_(PO_4_)_3_F (Yb:S-FAP). This suggests that it is easier to control the orientation of Yb:FAP than that of Yb:S-FAP, and the alignment of the main crystal axis (*c*-axis) of the microdomains made of Yb:FAP microcrystals requires a rotating magnetic field^21^.

As illustrated in Fig. 2, the orientation control of microdomains was carried out during slip-casting of a slurry containing Yb:FAP microcrystals with a rotating casting mold under ***B*** before sintering. The theoretical limit of the mean deflection angle <*θ*> between the *c*-axis and controlling direction using the rotating magnetic field ***B*** is approximately given by

4 $$\langle \theta \rangle ={\mathrm{Sin}}^{-1}\sqrt{\frac{24{\mu }_{0}kT}{36{\mu }_{0}kT+\pi {\delta }^{3}\Delta \chi {|{\boldsymbol{B}}|}^{2}}},$$

where *δ* is the diameter of microdomains. In this work, *Δχ* was assumed to be −4.6 × 10^−6^. *B* was 1.4 T generated by a traditional electromagnet, and the mean diameter of primary particles in the slurry *δ* was *ca*. 300 nm. Equation (4) reveals that we can realize <*θ*> of at least 21.6° when *δ* is 300 nm. If only the orientation of microcrystals with *δ* larger than 1.0 μm occurs because of preferential grain growth based on Ostwald ripening^20^, we can expect that <*θ*> is smaller than 5.8°.

**Figure 3:**
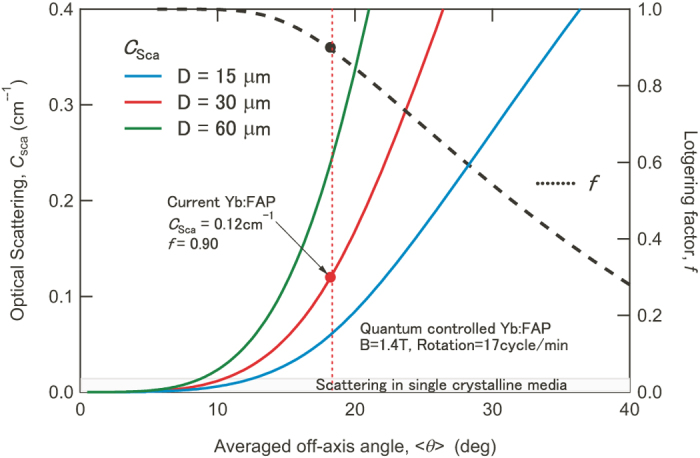
The relationships between <*θ*>, *f* and *C*_sca_ in Yb:FAP with quantum-controlled microdomains. <*θ*>, *f*, and *C*_sca_ are the theoretical mean deflection angle between the *c*-axis and controlling direction, the Lotgering factor, and the optical scattering coefficient. Dotted and solid lines show the relations between <*θ*> and *f*, and between <*θ*> and *C*_sca_, respectively.

Although the Lotgering factor *f* with a range from 0 (uncontrolled) to 1 (perfectly controlled) is used as a degree of the alignment in traditional discussions of orientation control^22^, we cannot directly consider optical scattering using *f*. Therefore, we have to convert *f* to the optical scattering coefficient *C*_sca_. We already developed a method to estimate <*θ*> from *f* ^23^, and the refractive index of a FAP crystal has also been reported^23^. The relationships between <*θ*>, *f* and *C*_sca_ calculated for Yb:FAP are depicted in Fig. 3.

Our Yb:FAP sample with QC-MDs displayed *f* of 0.9^23^, and <*θ*> of our Yb:FAP sample is 18° as shown in Fig. 3. If the mean diameter of micrograins *D* is 30 μm, *C*_sca_ of 0.12 cm^−1^ caused by birefringent optical scattering still remains in our Yb:FAP sample. Even though the calculation based on Equation (4) implies we have achieved process control, we can greatly lower *C*_sca_ to the same level as that of single-crystalline materials through well-controlled preferential grain growth. In any case, this analysis suggests that giant laser output pulses from Yb:FAP with QC-MDs should be realized when we can generate larger optical gain in it than 0.12 cm^−1^.

### Appearance of synthesized Yb:FAP

**Figure 4:**
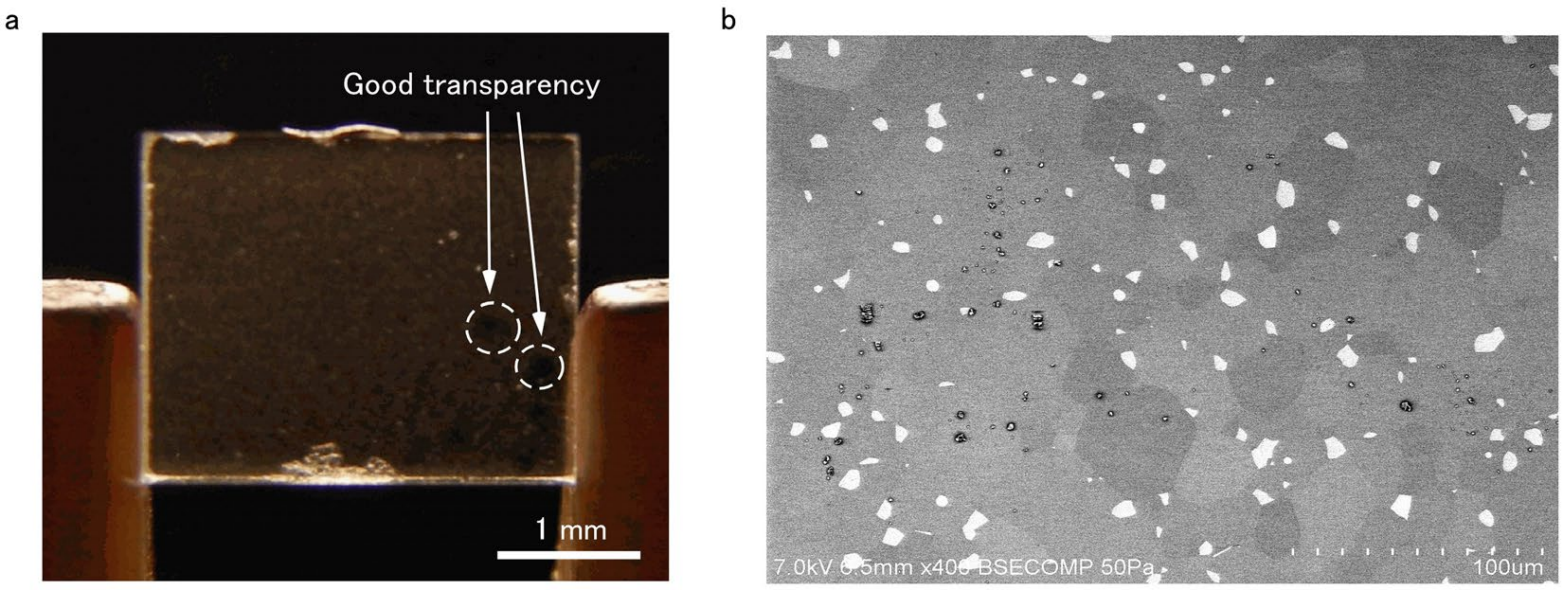
(**a**) Photograph and (**b**) SEM image of Yb:FAP with quantum-controlled microdomains.

The scattering sources are clearly shown in Fig. 4(a), where they were visualized by irradiation with white light and enhanced by the contrast with the black background. Figure 4(a) reveals that the main component of scattering is caused not by birefringence, but rather other point sources. Also, Fig. 4(a) shows that there are some transparent spots with a diameter of *ca*. 100 µm that display less scattering than the other areas of the sample. Scattering loss of 0.12 cm^−1^ should be realized in these transparent spots, where easier laser oscillation can be expected than in other parts of the sample.

To further investigate these point scattering sources, a backscattered electron image obtained using a scanning electron microscope (SEM) is presented in Fig. 4(b). There are two types of contrast in this SEM image: one is a high contrast that is caused by the difference in the elemental composition of the sample, and the other is a low contrast that originates from the difference in crystal orientations. The high-contrast regions in the matrix should be amorphous phases, because there were only peaks consistent with FAP in the X-ray diffraction pattern^21^. The low-contrast structures were able to be observed as a result of fine alignment of the SEM. These structures consisted of microdomains in Yb:FAP with well-controlled orientations and a mean diameter of *ca*. 30 μm.

### Laser experiments

**Figure 5:**
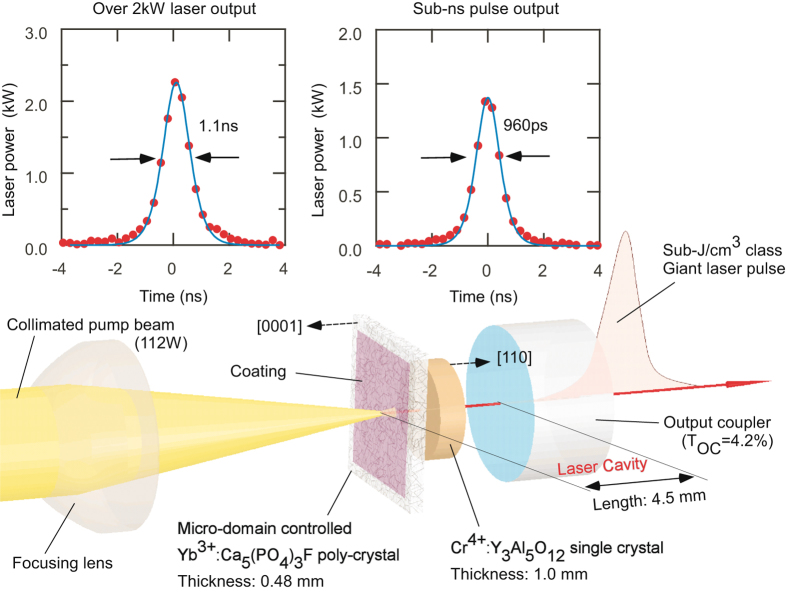
Experimental setup and generated giant pulses of the laser oscillation in Yb:FAP with quantum-controlled microdomains.

To prove the formation of Yb:FAP with QC-MDs, we tried to obtain high output power through passive Q-switching of the MCL, as illustrated in Fig. 5. When the pumped area in Yb:FAP was tuned to have high peak power, a maximum peak power of 2.3 kW was obtained. Yb:FAP generated pulse trains with a pulse duration of 1.1 ns at 983 nm 255 µs after the start of excitation by a pump laser diode. The total output energy was 8.53 µJ in four pulses with a mean interval of 124 µs. By tuning the focal spot to where the sample produced the minimum pulse duration, Yb:FAP generated sub-ns pulses with a 1.4-kW peak power; that is, giant pulses in the “pulse-gap” region^2^. Pulses in these trains should have polarization perpendicular to the *c*-axis of Yb:FAP because the laser beam passed across the sample along the *c*-axis. To keep the lasing, the center of the focal point of the pump beam had to be limited within an area with a diameter of 130 µm, which is consistent with the distribution of optical scattering in Fig. 4(a). Thus, these laser outputs were extracted only within this range, even though the pumped spot had a diameter of 0.4 mm. Clear apertures in Fig. 4a had a spotsize of 130 µm and a thickness of 480 µm. Therefore, it is natural to consider that the size of clear apertures relates not to the size of microdomains but to the density of the amorphous phase.

## Discussion

**Figure 6:**
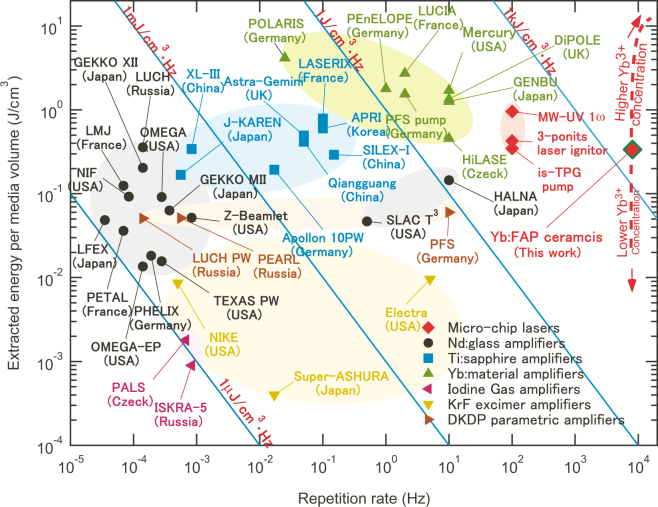
The figure of merit of G-MiP and representative high-power laser devices.

As mentioned above, both the repetition rate and extraction energy density are appropriate for the figure of merit (FOM) of G-MiP. The experimental results indicate that the FOM of the current Yb:FAP sample with QC-MDs includes a repetition rate of 8.1 kHz and extraction energy density of 0.34 J/cm^3^. Figure 6 compares the FOM for representative high-power laser devices. It should be noted that Fig. 6 indicates not the performance of existing laser devices, but rather the future possibility of power scaling based on G-MiP. As a result of selection of FOM parameters, Fig. 6 also shows the evolution of developments of solid-state laser gain media. The first material group is Nd^3+^-doped glasses with high capacity for energy storage^24^, the second group is Ti:sapphire lasers with high thermal conductivity and the capability for chirped pulse amplification (CPA)^25^, and the third group is Yb^3+^-doped media with both high energy capacity and CPA ability^26^. Even though there are still many problems to be overcome before practical usage of G-MiP, Fig. 6 leads us to believe in the possibility of G-MiP technology exemplified by Yb:FAP.

When using Yb^3+^-doped media, it is necessary to overcome their small *σ*_e_, which is currently achieved by cryogenic operation at around 200 K^27, 28^. Therefore, the large *σ*_e_ of our Yb:FAP with QC-MDs needs to be confirmed to clarify its advantage over other Yb^3+^-doped media. The experimental laser output energy and timing indicated that *σ*_e_ of our Yb:FAP was 3.6 × 10^−20^ cm^2^. This value is 1.7 times larger than that of Yb:YAG at room temperature, and comparable to *σ*_e_ of cryogenically operated devices. Although *σ*_e_ was only 40% of that reported for single-crystalline Yb:FAP in *σ*-polarization^23^, this situation implies that we can expect 1.4 times larger *σ*_e_ than current Yb:FAP with QC-MDs by future improvements in crystal quality. Moreover, the fabrication of larger bulk devices made of Yb:FAP with QC-MDs will allow us to realize effective *σ*_e_ of 8 × 10^−20^ cm^2^ like *π*-polarization in Yb:FAP single crystals.

The extraction energy density can be increased by obtaining higher inversion density with higher Yb^3+^ doping. Using ceramic technology that allows the RE^3+^ concentration of laser gain media to be increased compared with that of single-crystalline media^17^, future power scaling of Yb:FAP with QC-MDs will be realized based on the increase of both of the extraction energy density and repetition rate, as indicated by the red dashed line in Fig. 6. Besides higher FOM, greater Yb^3+^ doping will also enhance pump absorption efficiency and *Δχ* to realize more precise orientation control, as described in Equation (4). Because 2 kW-level power enables two-photon excitation, Yb:FAP with QC-MDs is a candidate for *in vivo* laser gain media^29^. For example, the oscillation wavelength of 983 nm in this work is suitable as a pumping source for two-photon excitation of channelrhodopsin-2 to control brain activity^30^. As indicated in previous section, the current obstacles limiting power scaling of our devices is the existence of the amorphous phase (Fig. 4(b)). We think it was caused by the partial melting of Yb:FAP due to the variation in Yb^3+^-concentration, and we are now trying to eliminate this secondary phase by improving the Yb^3+^-doping process.

From the viewpoint of the industrialization of G-MiP, the elimination of superconducting magnets from the fabrication process is preferable. Even though we limited magnetic field to 1.4 T, we realized orientation-controlled polycrystals with *f* of 0.9, indicating that the microdomains in our sample were almost perfectly controlled. Thus, superconducting magnets are not always necessary to control the orientation of diamagnetic materials.

Traditional translucent anisotropic ceramics such as transparent alumina achieve transparency by possessing smaller grain sizes than the wavelength of visible light^16^. Instead, we aligned the orientation of the crystal axes in microdomains to maximize the benefits of anisotropic optical characteristics. Here, Yb:FAP with a mean domain size of 30 μm showed enough transparency to allow laser oscillation. This reveals that QC-MDs are another solution to small grain size to achieve transparent ceramics.

Recently, birefringent optical scattering in laser ceramics has been suppressed only by microdomains with cubic crystalline symmetry^17^. The maximum output power of laser action inside anisotropic polycrystalline materials before this work was hundreds of milliwatts^31^. The kilowatt-level output power and future power scalability proposed in this work will allow use of crystals with non-cubic crystalline symmetry as raw materials of large-sized polycrystalline bulk media for high-power lasers. Of course, it should be possible for Nd:FAP in ref. 31 to realize several-kW output by use the same setup. However, the large quantum defect and the heavy concentration quenching prevent to apply our solution for the future power-scaling to Nd^3+^-doped gain media. Thus, Yb:FAP with QC-MDs should be the best candidate even though the process control of Nd:FAP with QC-MDs are easier than Yb:FAP.

We realized the concept of QC-MDs as an important technology in G-MiP. Before this work we were able to expect only the sign of the magnetic anisotropy induced by RE^3+^, and it had been impossible to evaluate whether the orientation controls of microdomains were effectively processed or not. This work offers the quantitative method how to design the magnetic anisotropy induced by various kinds of rare-earth ions and how to design the orientation distribution for crystalline axes of microdomains. As we demonstrated by use of Yb:FAP in this work, people can design the magnetic anisotropy by means of our procedure in this work. G-MiP using both MCL and QC-MD technologies represents a new frontier of highly bright optical sources suitable for implementation in living bodies to realize brain–machine interfaces and laser therapy.

## Methods

### Electron cloud of the 4*f*-hole in Yb^3+^ under a crystal field

In Fig. 1, the state of Yb^3+^ can be described as eigenstates of the crystal field potential, and is composed of |*J*, *J*_z_> = (*J*_−_)^m^ *Y*_33_(*θ*, *ϕ*)*χ*_↑_ (*m* = 0 to 7), where *Y*_*lm*_(*θ*, *ϕ*), *J*_−_ and *χ*_↑_ are spherical harmonics, the ladder operator of total angular momentum and the wave function of spin with +0.5 *ħ*, respectively. The crystal field potentials for the crystals were calculated using the Stevens equivalent operator as in Equation (2)^19^. We can approximate the radius of the electron cloud in the direction of angle (*θ*, *ϕ*) by the azimuthal component to the 1.5^th^ power. Electron clouds in this viewgraph were drawn larger than the real size to improve visibility.

### Calculation and experimental measurement of *Δχ*

Here we show how to estimate *Δχ* of RE^3+^-doped microdomains. In order to calculate the effective total angular momentum of doped RE^3+^-ions, we have to introduce two new multipliers. One describes Coulomb shielding, and it is a ratio of the calculated *V*_cryst_ to the spectroscopically measured *V*_cryst_. Another describes Néel rotation of doped RE^3+^-ions^32^, which can be estimated from the ratio of the calculated *Δχ* to the measured *Δχ* in the other host crystal where the same RE^3+^-ions are doped.

Using a superconducting quantum interference device magnetometer under 7.0 T (MPMS-7, Quantum Design Inc., San Diego, CA, USA) in the temperature range from 80 to 300 K, we measured *Δχ* of Yb^3+^-doped and undoped yttrium orthovanadate (YVO_4_) single crystals with dimensions of 4.0 × 4.0 × 3.0 mm, and S-FAP with dimensions of 3.5 × 3.5 × 3.5 mm (Fig. 2). Yb^3+^ are substituted into 4*a* sites in YVO_4_ and 6 *h* sites in apatite crystals. According to Equation (2), *V*_cryst_ in YVO_4_, S-FAP and FAP were evaluated using lattice sums that were calculated with over 30,000 nearest ions around the doped sites for the crystals. *V*_cryst_ was multiplied by a constant to correct the crystal field splitting modulated by Coulomb shielding of RE^3+^. Mainly because of Néel rotation of the magnetic moments, the measured *Δχ* became smaller than the theoretical value determined by Equation (3). We estimated this factor to be 20% from the difference between calculated and experimental values for Yb:YVO_4_. Generally, undoped crystals show non-zero *Δχ* because of Larmor diamagnetism; *Δχ* was measured to be 3.9 × 10^−6^ for the YVO_4_ crystal. We estimated that *Δχ* for undoped S-FAP was 2.4 × 10^−6^ from the temperature dependence of the calculated and measured *Δχ* of Yb:S-FAP.

While the main crystal axis (*c*-axis) of Yb:S-FAP and Yb:FAP is the hard magnetization axis (*Δχ* < 0), the main crystal axis of Yb:YVO_4_ is the easy magnetization axis (*Δχ* > 0). This difference arises from the sign of their lattice sum. Similarly, the main crystal axis of Nd^3+^-doped apatite crystals is the easy magnetization axis because of the different signs of *θ*_*J*_^33^.

### Calculation of *C*_s__c__a_

In uniaxial materials, refractive index *n*(*θ*) is given by

5 $$n(\theta )=\sqrt{{{n}_{s}}^{2}{\sin }^{2}\theta +{{n}_{m}}^{2}{\cos }^{2}\theta ,}$$

where *n*_m_ and *n*_s_ are refractive indices of the microdomains for polarizations that are parallel and perpendicular to the controlled axis, respectively. The mean refractive index of all domains *n*_mean_ and the difference of refractive index between the averaged matrix and each microdomain *Δn* are calculated from the distribution function *F*(*θ*) by

6 $${n}_{mean}={\int }_{0}^{\tfrac{\pi }{2}}F(\theta )n(\theta )\sin \,\theta d\theta ,$$

7 $${({\Delta }n)}^{2}={\int }_{0}^{\tfrac{\pi }{2}}F(\theta ){[n(\theta )-{n}_{mean}]}^{2}\,\sin \,\theta d\theta ,$$

In the case of Yb:FAP, wavevector ***k*** of the irradiated light is parallel to the *c*-axis. Consequently, *n*_m_ and *n*_s_ are 1.622 and 1.620, respectively^23^. *F*(*θ*) can be derived from Equation (1) as

8 $$F(\theta )=\frac{2B}{\sqrt{\pi }{B}_{\min }{\rm{Erfi}}(B/{B}_{\min })}\exp (\frac{{B}^{2}}{{{B}_{\min }}^{2}}{\cos }^{2}\theta ),$$

where Erfi(z) gives the imaginary error function, and *B*_min_ is a parameter that indicates the lower limit of the magnetic flux density for orientation control^20^. *B*_min_ is proportional to the square root of RE^3+^ concentration. *C*_sca_ arising from this birefringent scattering is expressed by^16^

9 $${c}_{sca}=\frac{3{{\pi }}^{2}D}{2{{\lambda }}^{2}}{\Delta }{n}^{2},$$

where *λ* is the wavelength of the incident light.

Although Equation (9) gives *C*_sca_ of 0.12 cm^−1^ for *D* of 30 μm, spectroscopic measurements indicated *C*_sca_ was 1.0 cm^−1^, which is eight times larger than the calculated value^21^. This difference suggests that there are other scattering sources in our Yb:FAP as well as birefringent scattering.

### Sample preparation

Some of the Ca^2+^ in FAP microcrystals were substituted by Yb^3+^ by reacting commercial FAP powder (10 g) and Yb(NO_3_)_3_ (1.5 g) in an aqueous solution (120 mL) containing 0.012 mol of hydrochloric acid and 0.012 mol of sodium nitrate for 5 min at room temperature. After drying, Yb:FAP microcrystals were dispersed in distilled water as a slurry for casting. As described in Fig. 2, slurry in a gypsum mold was rotated at 17 rpm, and slip-cast under a magnetic field of 1.4 T generated by an electric magnet (JER-3XG, JEOL Ltd., Tokyo, Japan). Cast powder compacts were pre-sintered for 2 h at 1600 °C in air, and hot isostatic pressed at 1600 °C and 190 MPa in Ar for 1 h (System 20 J, Kobe Steel, Ltd., Tokyo, Japan). The Yb:FAP sample was cut and polished to dimensions of 3.4 × 3.0 × 0.48 mm (Lambda Precision Co., Atsugi, Japan).

Quantum control under a magnetic field was a stochastic process that could be reproduced statistically if isolated microcrystals were well stabilized in the slurry used for the slip-casting process. A rough indication of mean deflection angle <*θ*> is given by

10 $${\sin }^{2}\langle \theta \rangle \approx {\int }_{0}^{\tfrac{\pi }{2}}F(\theta ){\sin }^{3}\theta d\theta \approx \frac{2{{B}_{\min }}^{2}}{3{{B}_{\min }}^{2}+2{B}^{2}}.$$

In the orientation control of Yb:FAP domains, the alignment of one hard magnetization axis is equivalent to the alignment of two easy magnetization axes. Consequently, *B*_min_ for Yb:FAP domains should be

11 $${B}_{\min }=2\sqrt{\frac{6{\mu }_{0}kT}{\pi {\delta }^{3}|\Delta \chi |}},$$

which is √2 times compared to the orientation control of one easy magnetization axis^20^. Equation (4) can be directly derived from Equations (10) and (11). However, we only managed to synthesize two samples of polycrystalline Yb:FAP with QC-MDs that displayed laser oscillations. The consistency of material processes in this work relies on the reproducibility of current laser ceramic technologies such as casting, sintering and polishing.

### Photographs and SEM

Figure 4(a) was taken under irradiation with white light to emphasize the optical scattering of the sample. To prevent charge-up on the surface of uncoated Yb:FAP, a back-scattered electron image was obtained by SEM (SU6600, Hitachi Ltd., Tokyo, Japan) under a vacuum of 50 Pa with a 7.0-kV accelerating voltage and 400× magnification (Fig. 4(b)). There were almost no structures in a SEM secondary electron image. Observation of the image in Fig. 4(b) indicates that the *C*_sca_ of 1.0 cm^−1^ in ref. 21 should be caused by both birefringence and the secondary amorphous phase.

### Laser experiments

Two surfaces of a Yb:FAP sample with an area of 3.4 × 3.0 mm were coated with coating apertures of 2.5 × 2.5 mm (Showa Optronics Co. Ltd., Tokyo, Japan): one side had reflection below 1% at 905 nm and above 99.9% around 1 µm (A-side), and the other side had reflection above 99.5% at 905 nm and below 0.5% around 1 µm (B-side). The laser resonator with a cavity length of 4.5 mm was composed of the A-side of Yb:FAP as a total reflection mirror and plane mirror with a reflection *R*_OC_ of 95.8% as an output coupler. The B-side of Yb:FAP was placed on a copper holder, and fixed from the A-side by a copper plate. A Cr^4+^:YAG film with a thickness of 1 mm, initial transmittance *T*_0_ of 80% and covered on both surfaces with anti-reflection coating (Scientific Materials Co., Boseman, MT, USA) was inserted as a saturable absorber between the output coupler and B-side of Yb:FAP. The pump source was a fiber-coupled laser diode with an output power of 112 W at 905 nm, duration of 800 µs and rise time of 20 µs (LE0379, Hamamatsu Photonics K.K., Hamamatsu, Japan). This pump source was collimated by an aspheric condenser lens with a focal length of 18 mm, and focused onto the A-side of Yb:FAP by an aspheric condenser lens with a focal length of 12 mm. Generated laser output passed across the pump-cut filter with 86.3% transmittance around 1 µm and was detected by an energy sensor (PD10-C, Ophir Optics LLC, North Andover, MA, USA) or photodetector (ET-3500F, Electro-Optic Technology Inc., Traverse City, MI, USA) and spectrum analyzer (AQ6763, Yokogawa Meters & Instruments Co., Tokyo, Japan).

### High-power laser facilities

In Fig. 6, National Ignition Facility (NIF), Laser Mégajoule (LMJ), PETawatt Aquitaine Laser (PETAL), LUCH (ISKRA-6), OMEGA, GEKKO, Z-Beamlet, Petawatt High Energy Laser for Heavy Ion Experiments (PHELIX), TEXAS, SLAC National Accelerator Laboratory (SLAC), Prague Asterix Laser System (PALS), Petawatt Field Synthesizer (PFS), JAEA Kansai Advanced Relativistic ENgineering (J-KAREN), Apollon, Qiangguang, SuperIntense Laser for Experiments on eXtremes (SILEX), Xtreme Light III (XL-III), Astra-Gemini, Laserix, Advanced Photonics Research Institute (APRI), Petawatt Optical Laser Amplifier for Radiation Intensive experimentS (POLARIS), Petawatt, Energy-Efficient Laser for Optical Plasma Experiments (PEnELOPE) and Mercury are petawatt-class laser facilities^34^. PEARL^35^ is the current laser system at Exawatt Center for Extreme Light Studies (XCELS)^34^. ISKRA-5^36^ is an iodine laser that is the previous generation of LUCH. ELECTRA, NIKE, and Super-ASHURA are high-power excimer lasers^37^. LUCIA and Diode Pumped Optical Laser Experiment (DiPOLE) are Yb:YAG ceramic laser systems that were developed as a part of the HiLASE project^27^. Generation of ENergetic Beam Ultimate (GENBU)^28^ and High Average power Laser for Nuclear fusion Application (HALNA)^38^ are categorized as slab laser systems that have high thermal performance.

In these laser facilities, effective cooling of laser gain media is quite important, and the advantage of MCL technology has been proved by the possibility of face cooling of thin laser gain media in Mercury, LUCIA, DiPOLE, HALNA, GENBU and SLAC. Consequently, MCLs such as MW-UV^39^, multi-point laser ignitor^40^, and is-TPG^41^ can provide comparable optical energy densities to the above high-power devices aiming to realize inertial confinement fusion.

### Evaluation of *σ*_e_

The averaged pump intensity in effective mode volume *I*_p_ inside Yb:FAP was 278 kW/cm^2^ in our setup, where the number density of Yb^3+^ *N*_tot_ was 1.86 × 10^20^/cm^3^ = 1.62 at. % and the pump saturation intensity *I*_s_ was 13.9 kW/cm^2^. The time evolution of *ξ*, which is the ratio of *N* to *N*_tot_, is given by

12 $${{\tau }}_{{\rm{f}}}\frac{d{\xi }}{dt}=-{f}_{{\rm{1}}}+({f}_{{\rm{2}}}-{f}_{{\rm{3}}})\frac{{I}_{{\rm{p}}}}{{I}_{{\rm{s}}}}-(1+\frac{{f}_{{\rm{1}}}+{f}_{{\rm{3}}}}{{f}_{{\rm{1}}}}\frac{{I}_{{\rm{p}}}}{{I}_{{\rm{s}}}}){\xi }{,}$$

where *f*_1_, *f*_2_ and *f*_3_ are the fractional population ratios of the ground state, laser upper level and pumping levels, respectively. We estimated that *f*_1_, *f*_2_ and *f*_3_ were 0.827, 0.909 and 0.0128, respectively^23^. Because the effective pumping time was 235 µs, we calculated that *ξ* was up to *ξ*_i_ = 0.785. Therefore, the initial population inversion *N*_i_ in this work was calculated to be 1.46 × 10^20^/cm^3^. The number of Yb^3+^ that were totally excited into the upper manifold *N*_U_ can be expressed by

13 $$\frac{{N}_{U}}{{N}_{tot}}=\frac{1}{{2}^{\ast }}({f}_{{\rm{1}}}+{{\xi }}_{{\rm{i}}}),$$

where 2^*^ is the bottleneck factor and equals *f*_1_ + *f*_2_. In Yb:FAP, 2^*^ is calculated to be 1.74. Thus, we can conclude that 92.5% of Yb^3+^ were excited. According to Equation (12), the repetition rate of 8.1 kHz indicates that the final population inversion after pulsing *N*_f_ was 1.16 × 10^20^/cm^3^.

We can estimate the stimulated emission cross section *σ*_e_ of our Yb:FAP sample. From the balance between loss and gain, with a *C*_sca_ of 0.120 cm^−1^, the initial gain coefficient *g*_i_ of Yb:FAP can be calculated to be

14 $$2{g}_{i}l=-\,\mathrm{ln}({R}_{{\rm{OC}}})-2\,\mathrm{ln}({T}_{{\rm{0}}})+2l{C}_{{\rm{sca}}}=0.502,$$

Therefore, *σ*_e_ is given by

15 $${{\sigma }}_{e}=\frac{{g}_{i}}{{N}_{i}}=3.57\times {10}^{-20}{{\rm{cm}}}^{2}.$$

This value can be confirmed by the extraction energy density. Total extracted photon density *ΔN* is

16 $${\Delta }N=\frac{{N}_{{\rm{i}}}-{N}_{{\rm{f}}}}{{2}^{\ast }}=1.75\times {10}^{19}{{\rm{cm}}}^{-3}.$$

Therefore, the photon extraction efficiency *η*_ext_ and threshold inversion *N*_th_ are calculated by

17 $${{\eta }}_{{\rm{ext}}}=\frac{{\Delta }N}{{N}_{{\rm{i}}}}=\frac{{\Delta }N}{{{\xi }}_{{\rm{i}}}{N}_{{\rm{tot}}}}=12.0 \% ,$$

18 $${N}_{{\rm{th}}}\approx -\frac{{2}^{\ast }{{\eta }}_{{\rm{ext}}}{N}_{{\rm{i}}}}{\mathrm{ln}\,(1-{2}^{\ast }{{\eta }}_{{\rm{ext}}})}=1.30\times {10}^{20}{{\rm{cm}}}^{-3}.$$

Finally, we can obtain the output coupling efficiency *η*_OC_ and energy extraction density *E*_out_ by

19 $${{\eta }}_{{\rm{OC}}}=\frac{-\mathrm{ln}({R}_{{\rm{OC}}})}{2{N}_{th}{{\sigma }}_{e}l}=9.61 \% ,$$

20 $${{{\rm E}}}_{out}=h{\nu }{{\eta }}_{{\rm{OC}}}{\Delta }N=0.340{J/\mathrm{cm}}^{-3}.$$

The fact that the experimental *E*_out_ of 0.34 J/cm^3^ coincided with the calculated value proves the validity of the estimated *σ*_e_. In addition, *C*_sca_ of 0.12 cm^−1^ and *σ*_e_ of 3.6 × 10^−20^ cm^2^ were also confirmed experimentally from the strong relaxation oscillation observed under the condition without Cr^4+^:YAG using recently developed methods^42^.

### Power scaling by increasing Yb^3+^ doping

The dependence of *E*_out_ on *N*_tot_ can be calculated using *η*_ext_ as

21 $${{{\rm E}}}_{out}=h{\nu }{{\eta }}_{{\rm{OC}}}{{\eta }}_{{\rm{ext}}}{{\xi }}_{{\rm{i}}}{N}_{{\rm{tot}}}.$$

From Equations (14) and (18), *ξ*_i_ and *η*_ext_ are given as functions of *N*_tot_ by

22 $${{\xi }}_{{\rm{i}}}=\frac{1}{{N}_{tot}}(\frac{{C}_{{\rm{sca}}}}{{{\sigma }}_{e}}-\frac{\mathrm{ln}\,{R}_{{\rm{OC}}}}{2{{\sigma }}_{e}l}-\frac{\mathrm{ln}({T}_{{\rm{0}}})}{{{\sigma }}_{e}l}),$$

23 $${{\eta }}_{{\rm{ext}}}=\frac{1}{{2}^{\ast }}[1+\frac{{N}_{{\rm{th}}}}{{{\xi }}_{{\rm{i}}}{N}_{{\rm{tot}}}}{\rm{\Omega }}(-\frac{{{\xi }}_{{\rm{i}}}{N}_{{\rm{tot}}}}{{N}_{{\rm{th}}}}{e}^{-\frac{{{\xi }}_{{\rm{i}}}{N}_{{\rm{tot}}}}{{N}_{{\rm{th}}}}})],$$

where Ω(*x*) is the omega function (the inverse function of *xe* ^*x*^). The red dashed line in Fig. 6 indicates the power scaling calculated by Equation (23) assuming that *R*_OC_ had the same *ξ*_i_ as in our experiment of 0.785.
